# Adverse pregnancy outcomes among women presenting at antenatal clinics in Ouélessébougou, Mali

**DOI:** 10.1186/s12978-020-0890-7

**Published:** 2020-03-17

**Authors:** Naissem Andemel, Santara Gaoussou, Amadou Barry, Djibrilla Issiaka, Almahamoudou Mahamar, Moussa Traore, Patrick E. Duffy, Alassane Dicko, Michal Fried

**Affiliations:** 1 Laboratory of Malaria Immunology and Vaccinology, National Institute of Allergy and Infectious Diseases, National Institutes of Health, Bethesda, MD USA; 2 Malaria Research & Training Center, Faculty of Medicine, Pharmacy and Dentistry, University of Sciences Techniques and Technologies of Bamako, P.O Box 1805, Bamako, Mali

**Keywords:** Pregnancy, Miscarriage, Stillbirth, Neonatal death, Preterm delivery

## Abstract

**Background:**

In sub-Saharan Africa, malaria continues to scourge the population and is the primary cause of morbidity and mortality in young children and pregnant women. As current preventative measures such as intermittent preventive treatment and use of insecticide-treated nets provide incomplete protection, several malaria vaccines are currently under development, including one to specifically prevent pregnancy malaria. Prior to conducting vaccine trials, it is important to obtain background information on poor pregnancy outcomes in the target population to establish a baseline.

**Methods:**

Pregnant women presenting at community health care centers for antenatal care were recruited to the study. Gestational age was determined by ultrasound examination following recruitment. Antenatal care and pregnancy outcome information were collected during a visit 4–8 weeks post-delivery.

**Results:**

One thousand eight hundred fifty women completed the study, and analysis included 1814 women after excluding multiple gestations (*n* = 26) and missing/incomplete data (*n* = 10). The percentage (95% CI) of adverse pregnancy outcomes is as follows: miscarriage, 0.28% (0.04–0.52); stillbirth, 1.93% (1.30–2.56); early neonatal death, 1.65% (1.03–2.24); late neonatal death, 0.39%, (0.10–0.68); and preterm delivery (PTD), 4.74% (3.76–5.73). The percentages of early and late neonatal deaths and PTD were significantly higher (*p* < 0.01) in primigravid compared to multigravid women. In primigravidae, 3.1, 1.1 and 7.1% of pregnancies resulted in early neonatal death, late neonatal death and PTD, respectively, while these outcomes in multigravidae were 1.0, 0.1 and 2.7%, respectively. Major malformations were identified in 4 newborns.

**Conclusions:**

Low gravidity and young age predict perinatal death and PTD. The information collected here can be used as a baseline for adverse pregnancy outcomes in future vaccine trials in pregnant women.

## Plain English summary

Malaria is a global scourge causing over 200 million cases and 400,000 deaths in 2017, with the majority occurring in sub-Saharan Africa. Pregnant women and their offspring are particularly susceptible to malaria infection which causes poor pregnancy outcomes such as preterm delivery, low birth weight, and severe maternal anemia. Current preventive measures provide incomplete protection, thus, a vaccine to prevent pregnancy malaria is urgently needed. Several malaria vaccines are currently under development, including candidates specifically designed to prevent pregnancy malaria. However, these candidates must be tested in pregnant women to ensure that they are safe and effective. While pregnant women have historically been excluded from clinical trials that evaluate the efficacy of drugs or vaccines (primarily due to safety concerns for both mother and her child), this attitude has shifted in recent years. Before testing a malaria vaccine or other interventions in pregnant women, it is critical to obtain background information on poor pregnancy outcomes in the target population. These background rates can be used as a baseline to differentiate between outcomes associated with the intervention and those associated with malaria infection. In the current study, we collected this background information on pregnancy outcomes in women living in a high malaria transmission area of Mali, setting a foundation for testing interventions such as malaria vaccines in pregnant women.

## Introduction

In 2015, an estimated 2.6 million stillbirths occurred worldwide, with 98% of them occurring in developing countries [1]. Multiple factors have been associated with stillbirths including maternal age, non-communicable diseases, and infectious diseases like malaria, Group B *Streptococcus*, and syphilis particularly in sub-Saharan Africa [1–3]. Malaria infection also increases the odds for preterm delivery, low birthweight and intrauterine growth retardation [4]. Increased systemic inflammatory immune response to malaria infection has been associated with increased risk of both pregnancy loss (miscarriage and perinatal death) and preterm birth [5]. Preterm birth is one of the leading causes for neonatal and under-5 child mortality [6].

Neonatal death accounts for about half of the deaths in children under-five, with infectious diseases responsible for about 23% of neonatal deaths (reviewed in [7]). Maternal immunization is an effective strategy to protect the mother, fetus and/or the newborn, as has been shown with tetanus toxoid or influenza vaccines which protect both mothers and neonates (reviewed in [8, 9]). In the future, maternal immunization may be expanded to include new vaccines that are being developed against group B Streptococcus and Respiratory Syncytial Virus [9–11].

In sub-Saharan Africa, malaria continues to be the primary cause of morbidity and mortality in young children and pregnant women. According to a recent WHO report, malaria cases in 2017 amounted to 219 million resulting in 435,000 deaths, over 90% of which occurred in sub-Saharan Africa [12].

Women in malaria-endemic areas acquire resistance to malaria after years of exposure, but their susceptibility increases significantly during pregnancy, particularly the first. In high transmission areas, pregnancy malaria due to *Plasmodium falciparum* impacts both maternal and fetal health. *P. falciparum* infection during pregnancy is associated with increased maternal anemia, low birthweight, preterm delivery and stillbirth [13–15] .

The standard of care for pregnant women in malaria-endemic areas recommended by the World Health Organization (WHO) includes 1) preventive treatments with the antimalarial drug sulfadoxine/pyrimethamine (SP) at each scheduled antenatal care visit from the second trimester at least one month apart [termed intermittent preventive treatment (IPTp)], 2) prompt diagnosis and treatment of acute malaria with Artemisinin-based combination therapies (ACT) or quinine, and 3) use of insecticide-treated bed net (ITN). However, despite the previous guideline of 3 doses of IPTp throughout pregnancy, only 54% of pregnant women received at least 1 dose of IPTp and only 22% of women received at least 3 IPTp doses in 2017 [12]. In East and Southern Africa, IPTp with SP has lost its efficacy due to the spread of drug-resistant parasites [16, 17] resulting in increased parasite burden in the placenta, placental inflammation and increased risk of fetal anemia [16, 18]. Therefore, an effective vaccine for pregnancy malaria is needed.

Currently, two vaccine types are being considered for preventing pregnancy malaria. The first vaccine is based on targeting a protein expressed on surface of infected erythrocytes sequestering in the placenta [19]. The second, named PfSPZ Vaccine (radiation-attenuated sporozoites, a Sanaria whole malaria organism vaccine product), is not specific for pregnancy malaria and is intended to prevent blood-stage infection [20]. Ideally, the vaccine should be used in adolescent females prior to becoming pregnant. However, at this early stage of development, it is unknown if a boosting dose during pregnancy will be required*.*

Multiple factors complicate the introduction of new vaccines, including epidemiological data on specific disease burden and background on pregnancy outcomes in the target population [21–23].

In the current study, background information on pregnancy outcomes in women living in a malaria-endemic area with access to a public health care system was collected. This information can be used as a baseline for adverse pregnancy outcomes in future vaccine trials in pregnant women.

## Methods

### Human subjects and clinical procedures

The study was conducted in the district of Ouélessébougou, Mali, an area with high seasonal malaria transmission located approximately 80 km south of the capital city Bamako. Women were recruited during pregnancy among women presenting to both public and private health care centers for antenatal care in Ouélessébougou between February 2017 and May 2018. Pregnant women of any age and pregnancy stage were asked to participate in the study. Temporary residence in the area or women with conditions that could impair their ability to understand the study were the only exclusion criteria. Pregnant women or guardians (adolescents aged < 15 years) gave signed informed consent after receiving an oral explanation from a study clinician in their native language. The protocol and study procedures were approved by the Institutional Review Board of the National Institute of Allergy and Infectious Diseases at the US National Institutes of Health (ClinicalTrials.gov ID NCT02974608), and the Ethics Committee of the Faculty of Medicine, Pharmacy and Dentistry at the University of Bamako, Mali. The total number of women attending antenatal clinic during the enrollment period was available for 233 out of 363 enrollment days (64% of the time). During that period, 22% of women were not interested in the study and therefore not screened.

Gestational age was determined by ultrasound examination (Siui CTS-7700+ ultrasound scanner) following recruitment. Antenatal care and pregnancy outcome information were collected at 4–8 weeks post-delivery through interview, physical examination of the newborn and review of the medical records.

### Pregnancy outcomes

Miscarriage was defined as pregnancy ending at < 28 gestational weeks, stillbirth as a delivery of a non-viable baby at a gestational age of > 28 weeks, early neonatal death as death occurring within 7 days of birth and late neonatal death as death occurring between 8 and 28 days after birth. In the statistical analysis, perinatal death included stillbirth and early neonatal death, and pregnancy loss included cases of miscarriage, stillbirth and neonatal death. Preterm delivery was defined as birth prior to gestational age of 37 weeks. LBW was defined as a birth weight of < 2500 g. Pregnant mothers aged < 20 years were defined as adolescents.

### Statistical analysis

Singleton pregnancies were included in the analyses presented here. Proportion of adverse outcomes were estimated as numbers of the adverse outcomes divided by the number of singleton pregnancies with 95% CI. Chi-squared test and Fisher’s exact test were used to compare proportions of categorical variables. Logistic regression models were used to examine the relation between risk factors and pregnancy outcomes. Multivariate models included all covariates that were significantly related to pregnancy outcomes at a level of *p* < 0.1 in the univariate model.

Three adverse pregnancy outcomes were separately analyzed [perinatal death, preterm delivery (PTD) and low birth weight (LBW)]. Viable term deliveries served as the reference group in the logistic regression analyses of perinatal death and PTD, and normal birthweight of viable deliveries as the reference group in the logistic regression analysis of LBW.

Data analyses were performed using JMP Software version 14.0.0.

## Results

### Study population

The study population included 1814 (Table 1) women enrolled into the pregnancy registry study conducted in Ouélessébougou, Mali, after excluding from the analysis multiple gestations (*n* = 26), and missing/incomplete data (*n* = 10)**.** 24.9% of the women were primigravidae and 29.7% were < 20 years old. Most women (70.3%) were enrolled during the 2nd trimester, and 62.4% of women enrolled at women’s first antenatal clinic (ANC) visit. Gestational age at enrollment was similar between primigravid, secundigravid and multigravid women (mean (SD) 23.8 (7.3), 24.6 (7.7) and 24.7 (7.4), respectively, but significantly higher in grand multigravid women [mean (SD) 26.9 (6.6), *p* < 0.0001 compared to other groups]. Similarly, gestational age at enrollment was significantly higher (p < 0.0001) in women aged > 35 years compared to women aged *<* 20 and 20–35 years. The majority of women received at least one dose of SP-IPTp (96.8%), used ITN (92.7%) and received tetanus toxoid vaccine (97.6%).

**Table 1:** Study population (*n* = 1814)

Age	n (%)	Gestational age at enrollment, weeks Mean (SD)
< 20	538 (29.7)	23.8 (7.5)
20–35	1172 (64.6)	24.8 (7.5)
> 35	104 (5.7)	27.6 (5.9)
Gravidity		
Primigravid	455 (25.1)	23.7 (7.4)
Secundigravid	366 (20.1)	24.7 (7.8)
Multigravid (3–6 pregnancies)	783 (43.2)	24.6 (7.4)
Grand multigravida (> 6 pregnancies)	210 (11.6)	27.1 (6.5)
Number of ANC visits: mean (SD)	2.9 (1.2)	
SP-IPTp doses		
0	59 (3.3)	
1–2	1249 (68.9)	
> =3	506 (27.8)	
Used ITN	1681 (92.7)	

Laboratory tests were performed during antenatal visits as clinically indicated. 228 (12.6%) women had at least one documented malaria episode diagnosed with either blood smear or rapid diagnostic test. Hemoglobin levels were measured in 103 women (5.7%), 93 of which had a hemoglobin level of < 11 g/dL.

Other known maternal risk factors for PTD or perinatal death included hypertensive disorders of pregnancy (gestational hypertension, preeclampsia, eclampsia, and chronic hypertension), which were diagnosed in 11 women.

1786 of the 1814 women delivered at local health centers attended by trained midwives. Cases requiring cesarean section were referred to the district hospital. Delivery by cesarean section occurred in 2.7, 8.1, 5.7 and 6.6% of live births, preterm deliveries, stillbirths and early neonatal deaths, respectively.

### Pregnancy outcomes

Of the 1814 pregnancies, 5 resulted in miscarriage, 35 stillbirths, 37 neonatal deaths and 86 PTD (Table 2). Of the 35 stillbirths, 16 were macerated, 15 non-macerated and 4 cases not defined. The percentages of early and late neonatal deaths and PTD were significantly higher in primigravidae compared to multigravidae (Table 2). Similarly, when analyzed by age, the percentages of early and late neonatal deaths and PTD were significantly higher in adolescent mothers aged < 20 years (Table 2).

**Table 2:** Adverse pregnancy outcomes in the population (A), stratified by gravidity (B) and age (C)

A										
			n		% (95% CI)					
Miscarriage			5		0.28 (0.04–0.52)					
Stillbirth			35		1.93 (1.3–2.56)					
Early neonatal death			30		1.65 (1.03–2.24)					
Late neonatal death			7		0.39 (0.1–0.68)					
PTD			86		4.74 (3.76–5.72)					
**B**										
	Primigravid			Secundigravid			Multigravid		Grand multigravid	
	n	% (95% CI)	*P* value^1^	n	% (95% CI)	P value^1^	n	% (95% CI)	n	% (95% CI)
Miscarriage	2	0.4 (−0.2–1.1)	NS	1	0.27 (−0.26–0.8)	NS	2	0.26 (−0.1–0.3)	0	
Stillbirth	9	2.0 (0.7–3.3)	NS	7	1.91 (0.4–3.31)	NS	15	1.9 (1.0–2.9)	4	1.9 (0.1–3.8)
Early neonatal death	14	3.1 (1.5–4.7)	0.006	4	1.1 (0.03–2.2)	NS	8	1.0 (0.3–1.7)	4	1.9 (0.1–3.8)
Late neonatal death	5	1.1 (0.1–2.1)	0.02	0		NS	1	0.1 (−0.1–0.4)	1	0.5 (− 0.5–1.4)
PTD	36	7.1 (5.4–10.4)	< 0.0001	20	5.5 (3.1–7.8)	0.03	21	2.7 (1.6–3.8)	9	4.3 (1.6–7.0)
**C**										
	Age < 20			Age 20–35		Age > 35				
	n	% (95% CI)	P value^2^	n	% (95% CI)	n	% (95% CI)	P value^2^		
Miscarriage	2	0.4 (−0.1–0.9)	NS	3	0.3 (−0.03–0.6)	0		NS		
Stillbirth	11	2.0 (0.9–3.2)	NS	20	1.7 (1.0–2.5)	4	3.9 (0.2–7.6)	NS		
Early neonatal death	18	3.4 (1.8–4.9)	0.0009	12	1.0 (0.4–1.6)	0		NS		
Late neonatal death	5	0.9 (0.1–1.7)	0.01	1	0.09 (−0.08–0.3)	1	1.0 (− 0.9–2.8)	NS		
PTD	37	6.9 (4.7–9.0)	0.002	41	3.5 (2.5–4.6)	8	7.7 (2.6–12.8)	0.05		

^1^Fisher’s exact test in comparison to multigravid women

^2^Fisher’s exact test in comparison to women aged 20–35 yr

*NS* Not significant

The percentage of PTD was also higher in women aged > 35 years compared to women 20–35 years and the difference approached significance (*p* = 0.05).

Major malformations presented as brain anomaly (*n* = 3) and trisomy (*n* = 1) were documented among the live births. Minor malformations were reported in 67 newborns with the majority classified as umbilical hernia and skin abnormalities (Table 3).

**Table 3:** Malformation

	n
*Major malformations*	
Brain anomaly	3
Genetic (Trisomy)	1
*Minor malformations*	
Congenital abnormalities of hands and feet	5
Umbilical hernia	28
Skin (including birth marks, Mongolian spots and scars)	34

### Factors associated with pregnancy outcomes

The relationships between gravidity, age, malaria prevention [SP-IPTp and insecticide treated net (ITN)], delivery mode, and a composite of multiple risk factors were evaluated in univariate logistic regression analyses (Table 4). The composite of multiple risk factors consisted of those indicated in the ANC card (height < 150 cm, weight < 45 kg, history of cesarean section, miscarriage and stillbirth) named as “at least 1 known risk factor”. Primigravidae and women aged < 20 years had significantly higher odds of perinatal death compared to multigravidae and women aged 20–35 years. ITN usage reduced the odds of perinatal death, but this association did not achieve significance. The odds of PTD increased in primigravidae and secundigravidae. Adolescents, age > 35 years, and delivery by caesarian section were also associated with increased odds of PTD.

**Table 4:** Logistic regression analysis of maternal factors associated with perinatal death and PTD

	Univariate			Multivariate			
Outcome	Risk factor	OR (95% CI)	P value	AOR (gravidity) (95% CI)^1^	P value	AOR (age) (95% CI)^2^	P value
Perinatal death	Gravidity						
	Primigravid	1.89 (1.05–3.42)	0.03	1.85 (1.02–3.34)	0.04		
	Secundigravid	1.05 (0.51–2.19)	0.9	1.05 (0.50–2.17)	0.9		
	Multigravid	Reference					
	Grand multigravid	1.33 (0.59–3.03)	0.5	1.34 (0.59–3.04)	0.5		
	Age						
	< 20	2.13 (1.28–3.57)	0.004			2.09 (1.25–3.50)	0.005
	20–35	Reference					
	> 35	1.50 (0.52–4.35)	0.5			1.55 (0.54–4.48)	0.4
	> = 1 dose SP-IPTp	0.63 (0.19–2.08)	0.5				
	Used ITN	0.48 (0.21–1.08)	0.07	0.52 (0.22–1.13)	0.1	0.51 (0.22–1.15)	0.1
	ANC visits^3^	−0.01 (−0.20–0.19)	0.9				
	At least 1 known risk factor	1.17 (0.62–2.22)	0.6				
	Delivery mode^4^	2.34 (0.82–6.72)	0.1				
PTD	Gravidity						
	Primigravid	3.24 (1.87–5.63)	< 0.0001	3.25 (1.87–5.65)	< 0.0001		
	Secundigravid	2.10 (1.12–3.92)	0.02	2.19 (1.17–4.11)	0.01		
	Multigravid	Reference					
	Grand multigravid	1.64 (0.74–3.65)	0.2	1.68 (0.754–3.73)	0.2		
	Age						
	< 20	2.13 (1.35–3.36)	0.001			2.14 (1.35–3.39)	0.001
	20–35	Reference					
	> 35	2.35 (1.07–5.16)	0.03			2.32 (1.05–5.10)	0.04
	> = 1 dose SP-IPTp	0.85 (0.26–2.77)	0.8				
	Used ITN	1.18 (0.42–3.30)	0.7				
	ANC visits^3^	0.15 (−0.03–0.34)	0.1				
	At least 1 known risk factor	1.10 (0.66–1.82)	0.7				
	Delivery mode^4^	3.16 (1.38–7.24)	0.006	3.25 (1.40–7.56)	0.006	3.20 (1.39–7.38)	0.006

^1^AOR (Adjusted OR): Age is not included

^2^AOR (Adjusted OR): Gravidity is not included

^3^ANC visits: the number of ANC visits (coefficient and 95% CI)

^4^Delivery mode: cesarean section compared to spontaneous vaginal delivery (reference)

Because of the co-linearity between gravidity and age, separate multivariate logistic regression models were performed for evaluating their effects (Table 4). In this multivariate analysis, primigravidae or young age, remained significantly associated with increased odds of perinatal death. Primigravidae, secundigravidae, or women aged < 20 and > 35 years and those delivering by caesarian section had significantly increased odds of PTD in multivariate models.

Young adolescents (< 15 years) were at increased risk for adverse pregnancy outcomes compared to adolescents age 15–19 years [24]; in a secondary analysis, an age of < 16 years was evaluated (Supplementary Table 1). In this analysis, an age of < 16 years was selected because this age cutoff is treated as a risk factor in health centers of Mali. Similar to the multivariate analysis described above, age of < 16 years increased the odds of PTD [OR (95% CI) 6.07 (2.53–14.60)]. Age of < 16 years also increased the odds of perinatal death, but the relationship did not achieve statistical significance [OR (95% CI) 3.24 (0.95–11.08) *p* = 0.06].

Birthweight information was available for 1692/1737 live births (term and preterm delivery). 6.03% of live births were LBW. In a univariate logistic regression analysis, low gravidity (primigravidae and secundigravidae) or adolescence increased the odds of LBW (Table 5).

**Table 5:** Univariate logistic regression analysis of maternal factors associated with LBW

	OR (95% CI)	P value
Gravidity		
Primigravid	3.50 (2.14–5.73)	< 0.0001
Secundigravid	1.70 (0.92–3.05)	0.09
Multigravid	Reference	
Grand multigravid	1.17 (0.52–2.63)	0.7
Age		
< 20	2.31 (1.53–3.49)	< 0.0001
20–35	Reference	
> 35	1.19 (0.46–3.07)	0.7
> =1 dose SP- IPTp	0.71 (0.25–2.03)	0.5
Used ITN	0.76 (0.34–1.68)	0.5
ANC visits^1^	0.12 (−0.04–0.29)	0.2
At least 1 known risk factor	0.82 (0.46–1.46)	0.5
Delivery mode^2^	0.47 (0.19–1.12)	0.09

^1^ANC visits: the number of ANC visits (coefficient and 95% CI)

^2^Delivery mode: cesarean section compared to spontaneous vaginal delivery (reference)

### Malaria infection during pregnancy 

Women were not routinely tested for malaria infection. Malaria diagnosis by RDT or blood smear microscopy was extracted from the ANC card. Consistent with pregnancy malaria epidemiology, the percentages of primigravidae and secundigravidae diagnosed with malaria infection were higher than multigravid and grand multigravid women (Table 6). Being primigravidae or secundigravidae increased the odds of malaria infection diagnosis, OR (95% CI) 2.14 (1.53–2.99), *p* < 0.0001 and 1.74 (1.20–2.52), *p* < 0.003, respectively. Although the percentage of pregnancy loss was higher in primigravidae than multigravidae [7.1% vs 2.7% (Supplementary Table 2)], the difference did not achieve statistical significance (*p* = 0.2), possibly due to small sample size. Similarly, the percentage of pregnancy loss was higher in adolescent women compared to women aged 20–35, but the difference did not achieve significance (*p* = 0.1).

**Table 6:** Study subpopulation: malaria infection complaint (*n* = 228)

Age^1^	n (%)
< 20	100 (18.6)
20–35	120 (10.2)
> 35	8 (7.7)
Gravidity ^1^	
Primigravid	84 (18.5)
Secundigravid	57 (15.6)
Multigravid	75 (9.6)
Grand multigravid (> 6 pregnancies)	12 (5.7)
Number of ANC visits: mean (SD)	3.3 (1.3)
SP-IPTp doses^2^	
0	9 (3.9)
1–2	157 (68.9)
> =3	62 (27.2)
Used ITN	217 (95.2)

^1^Percent of total group in the study

^2^Percent of subpopulation

## Discussion

In this surveillance study conducted in an area with high seasonal malaria transmission, 4.2% of the pregnancies resulted in pregnancy loss (combined miscarriage, perinatal death and late neonatal death) mostly due to stillbirths and early neonatal deaths. Adverse pregnancy outcomes were analyzed in relation to multiple factors such as, maternal age, gravidity, number of ANC visits, malaria prevention (SP-IPTp and ITN), known risk factors and delivery mode. The main risk factors for these 3 adverse pregnancy outcomes included low gravidity (primigravid women) and young age. The Global Network’s Maternal Newborn Health Registry conducted in 7 countries (including 2 sites in sub-Saharan Africa) reported that overall early neonatal death rate was 20.6 per 1000 live births and neonatal death (during the first 28 days) was 25.7 per 1000 live births [25]. Major risk factors associated with neonatal death included PTD and LBW births [25]. In the same population, the average stillbirth rate was 28.9 per 1000 births [26]. Similar to neonatal death, increased risk of stillbirth was associated with PTD and LBW. Complicated delivery, male fetus, multiple gestation and congenital anomalies were additional risk factors of stillbirth [26]. Adolescents living in sub-Saharan Africa and Latin America were at a higher risk of pregnancy resulting in perinatal death compared to women aged 20–35 years [24]. In the current study, rates of stillbirths and neonatal deaths were similar to the reported rates in the Global Network’s Maternal Newborn Health Registry, and adolescence increased the odds for pregnancy loss. In the Global Network’s Maternal Newborn Health Registry, 67% of stillbirths were non-macerated, suggesting that a large proportion of stillbirths were associated with intrapartum complications [26]. Here, about 50% of the stillbirths were macerated, suggesting that half of stillbirths occurred due to other reasons prior to delivery.

In the current study, 4.7% of live births were preterm deliveries and 6.03% were LBW. The odds of PTD and LBW were significantly higher in primigravidae and secundigravidae compared to multigravidae. Because most of the primigravidae were < 20 years old, age was analyzed separately. Similar to previous findings in large pregnancy registry studies [24, 27], young maternal age was associated with increased odds of both PTD and LBW.

In this study, neither the number of ANC visits nor trimester of first ANC visit were associated with improved outcomes as reported in other studies. We speculate that this may be due to a large proportion of first visits occurring during the 2nd trimester, with a median gestational week of 21 for 73% of enrolled women; thus, we did not have enough power to evaluate this factor. The late attendance did not allow for capturing the rate of early miscarriage. To collect information on early pregnancy loss, non-pregnant child-bearing age women from the same population are currently enrolled and being followed up with monthly visits to capture pregnancy early on.

Low gravidity and maternal age predicted increased odds of LBW. A previous study conducted in Mali described that 2 doses of SP-IPTp reduced the odds of LBW [28]. In the current study, SP-IPTp did not reduce the odds of LBW when analyzed as a binary variable (Table 5) or as the actual number of doses. The lack of an apparent effect could be due to the fact that 96.8% of enrolled women received at least one dose of SP-IPTp.

The current study was based on extracting information recorded during antenatal visits and at delivery, in the antenatal card and a questionnaire. Because the study relies on data collected at health centers, information is missing regarding several variables known to contribute to adverse pregnancy outcomes. For example, malaria infection is diagnosed at the ANC in women presenting with clinical symptoms, resulting in history of a malaria infection available for only 228 women. In a cross-sectional survey of malaria infection in pregnant women living in the same area, 18% of malaria-infected women were symptomatic, suggesting that more women were likely to be infected with malaria than reported. Laboratory results for toxoplasma and syphilis infections were also available for a small proportion of the women, because at the time of the study, these tests were not routinely done at the antenatal clinics. Nonetheless, the study provides background information on perinatal death, PTD and LBW in women with access to public health care that can be used as a baseline in future vaccine trials.

## Conclusions

In this survey of pregnancy outcomes in women with access to public health care, adverse pregnancy outcomes were more common in first-time mothers. 6.6% of primigravidae compared to 3.3% in multigravidae experienced pregnancy loss (of which 76.6% were perinatal death). PTD was also more common in primigravidae (7.1% of live births compared to 2.7% in multigravidae). Most primigravidae were young, and similar to the analyses according to gravidity, adolescents experienced higher rates of both pregnancy loss and PTD. Future intervention trials in pregnant women can use the data described here to compare adverse pregnancy outcome rates in the trials to rates under normal conditions, particularly adverse events occurring during the 2nd and 3rd trimester of pregnancy.

## Supplementary information

**Additional file 1 Supplementary Table 1** Multivariate logistic regression analysis of maternal factors associated with pregnancy loss and PTD. AOR: Adjusted odds ratio. Multivariate analysis included variables that were significant in univariate analyses.

**Additional file 2 Supplementary Table 2** Pregnancy outcomes: women diagnosed with malaria (*n* = 228). ^1^Pregnancy loss (combined miscarriage, stillbirth and neonatal death)

## Data Availability

All data analysed during this study are included in this published article.

## Abbreviations

IPTp: Intermittent preventive treatment in pregnancy
ITN: Insecticide-treated bed net
LBW: Low birthweight
PTD: Preterm delivery
