# Supplementary material for: Adverse pregnancy outcomes among women presenting at antenatal clinics in Ouélessébougou, Mali
Source: Reprod Health. 2020 Mar 17;17:39. doi: 10.1186/s12978-020-0890-7 (PMC7077143; doi:10.1186/s12978-020-0890-7)
Supplement: Supplementary file 1 — Additional file 1 Supplementary Table 1 Multivariate logistic regression analysis of maternal factors associated with pregnancy loss and PTD. AOR: Adjusted odds ratio. Multivariate analysis included variables that were significant in univariate analyses. [file 12978_2020_890_MOESM1_ESM.docx]

**Supplementary Table 1. Multivariate logistic regression analysis of maternal factors associated with pregnancy loss and PTD**

| Outcome | Risk factor | AOR (95% CI) | P value |
| --- | --- | --- | --- |
| Perinatal death | Age <=16 | 3.24 (0.95-11.08) | 0.06 |
|  | Used ITN | 0.48 (0.21-1.09)) | 0.08 |
| PTD | Age <=16 | 6.07 (2.53-14.60) | <0.0001 |
|  | Delivery mode | 3.21 (1.39-7.40) | 0.006 |

AOR: Adjusted odds ratio. Multivariate analysis included variables that were significant in univariate analyses.
