# Supplementary material for: Adverse pregnancy outcomes among women presenting at antenatal clinics in Ouélessébougou, Mali
Source: Reprod Health. 2020 Mar 17;17:39. doi: 10.1186/s12978-020-0890-7 (PMC7077143; doi:10.1186/s12978-020-0890-7)
Supplement: Supplementary file 2 — Additional file 2 Supplementary Table 2 Pregnancy outcomes: women diagnosed with malaria (n = 228). 1Pregnancy loss (combined miscarriage, stillbirth and neonatal death) [file 12978_2020_890_MOESM2_ESM.docx]

**Supplementary Table 2. Pregnancy outcomes: women diagnosed with malaria (n=228)**

|  | Pregnancy loss^1^  n (%) | PTD  n (%) | Term  n (%) |
| --- | --- | --- | --- |
| Gravidity |  |  |  |
| Primigravid | 6 (7.1) | 3 (3.6) | 75 (89.3) |
| Secundigravid | 2 (3.5) | 3 (5.3) | 52 (91.2) |
| Multigravid | 2 (2.7) | 3 (4.0) | 70 (93.3) |
| Grand multigravid | 1 (8.3) | 0 (0) | 11 (91.7) |
| Age |  |  |  |
| <20 | 7 (7.0) | 5 (5.0) | 88 (88.0) |
| 20-35 | 3 (2.5) | 4 (3.3) | 113 (94.2) |
| >35 | 1 (12.5) | 0 (0) | 7 (87.5) |

^1^Pregnancy loss (combined miscarriage, stillbirth and neonatal death)
